# The Large Scale Structure of Human Metabolism Reveals Resilience via Extensive Signaling Crosstalk

**DOI:** 10.3389/fphys.2020.588012

**Published:** 2020-12-16

**Authors:** Laura Gómez-Romero, Karina López-Reyes, Enrique Hernández-Lemus

**Affiliations:** ^1^Computational Genomics Division, National Institute of Genomic Medicine, Mexico, Mexico; ^2^Centro de Ciencias de la Complejidad, Universidad Nacional Autónoma de México, Mexico, Mexico

**Keywords:** metabolic networks, pathway crosstalk, functional redundancy, resilience, human metabolism

## Abstract

Metabolism is loosely defined as the set of physical and chemical interactions associated with the processes responsible for sustaining life. Two evident features arise whenever one looks at metabolism: first, metabolism is conformed as a very complex and intertwined construct of the many associated biomolecular processes. Second, metabolism is characterized by a high degree of stability reflected by the organisms resilience to either environmental changes or pathogenic conditions. Here we will investigate the relationship between these two features. By having access to the full set of human metabolic interactions as reported in the highly curated KEGG database, we built an integrated human metabolic network comprising metabolic, transcriptional regulation, and protein-protein interaction networks. We hypothesized that a metabolic process may exhibit resilience if it can recover from perturbations at the pathway level; in other words, metabolic resilience could be due to pathway crosstalk which may implicate that a metabolic process could proceed even when a perturbation has occurred. By analyzing the topological structure of the integrated network, as well as the hierarchical structure of its main modules or subnetworks, we observed that behind biological resilience lies an intricate communication structure at the topological and functional level with pathway crosstalk as the main component. The present findings, alongside the advent of large biomolecular databases, such as KEGG may allow the study of the consequences of this redundancy and resilience for the study of healthy and pathological phenotypes with many potential applications in biomedical science.

## 1. Introduction

Metabolism is defined as the sum of physical and biochemical processes in living organisms that either produce or consume energy. Metabolic alterations often lead to cellular dysfunction, which is usually translated into disease (DeBerardinis and Thompson, 2012). Metabolism and disease are so tightly linked that diseases associated with adjacent metabolic reactions present higher comorbidity than diseases that have no metabolic links between them (Lee et al., 2008). Also, driver reactions, defined as the smallest set of reactions that must be controlled to control the activity of all reactions of the metabolic network, have been proposed as potential therapeutic targets in cancer cells (Basler et al., 2016). Understanding how the metabolism works, is one of the foundations to understand human disease.

The metabolism can be studied through the study of the relationships between cellular processes which are defined by the metabolic pathways. A pathway is composed of a set of molecules; either all proteins, i.e., enzymes, transporters, transcription factors, and signaling proteins, or all metabolic reactions, i.e., compounds and enzymes, that are involved in a cellular process. This representation has been used to find organizational principles around different cellular processes (Guimera and Amaral, 2005) or to highlight differentially regulated pathways associated with disease (Schramm et al., 2010).

There are different kinds of biological networks commonly used to study specific types of molecular interactions. *Metabolic networks* are used to study all metabolic reactions. *Protein-protein interaction networks* represent all physical interactions between proteins. And *transcriptional regulatory networks* are employed to study the regulation between transcription factors and target genes (Costa et al., 2008).

Common topological properties have been characterized for all of these biological networks in different organisms and even across several kingdoms of life. The connectivity distribution for all three kinds of networks usually follows an approximate power law (Jeong et al., 2000; Yu et al., 2004; Ouma et al., 2018) which implies that they can be considered as scale-free networks. Scale-free networks have been shown to manifest *small world* network behavior, i.e., any node can be reached from any other node in a small number of steps. Also, scale-free networks have been shown to be tolerant to error, but vulnerable to direct attacks, i.e., the network breaks out when a small fraction of the most relevant nodes is removed from the system but the structure is very stable to very high levels of random mutations (Albert et al., 2000).

Integrated approaches to study system level biological functions, encompassing metabolic, signaling and regulatory networks have been developed. Some successful efforts were initially directed toward model organisms, in particular yeast and bacteria. A work from Palsson's group (Herrgård et al., 2006) consisted in the integration of the transcriptional regulatory and metabolic networks from *Saccharomyces cerevisiae*. Careful curation of the literature was used to reconstruct the transcriptional regulatory network behind nutrient metabolism, which was coupled with an already curated global scale metabolic network. This approach allowed the authors to predict changes in gene expression in response to perturbations. Further integrative studies continue to be developed, Covert et al. (2008) presented a comprehensive metabolic, transcriptional regulatory and signal transduction modeling scheme in *Escherichia coli*. Their approach was based on an extension of flux balance analysis. More recently, the work by Price and his group (Ma et al., 2015) applied a probabilistic model to study the metabolic and gene regulatory networks in *Mycobacterium tuberculosis*, without including explicitly the role of signaling pathways or supramolecular protein-protein interaction networks.

In the case of human metabolism, a study presented a complete reconstruction of a human metabolic network (Duarte et al., 2007). Such full metabolic model was used to formulate detailed computational models leading to specific predictions not only in metabolic activity, but also in gene expression activity. However, no detailed transcriptional regulatory network for human phenotypes was available for integration yet. The work by Kierzek's group integrated metabolic, transcriptional regulatory and signal transduction networks in specific contexts for some human cell types (bile acid homeostasis in human hepatocytes) (Fisher et al., 2013). Some recent works have integrated the role of metabolic pathways with other layers of biological regulation (Guo et al., 2018; Ravikrishnan et al., 2018). However, most integrative efforts have been circumscribed to comprehensive metabolic mapping (Cottret et al., 2018; Ravikrishnan et al., 2018; Shen et al., 2019). Some integrated approaches have been also developed in the context of particular phenotypes and diseases (Pirhaji et al., 2016; Bidkhori et al., 2018; Krishnan et al., 2018; Pandey et al., 2020).

Molecular interactions and functional associations are systematically stored in specialized databases such as *STRING* (Szklarczyk et al., 2019), *REACTOME* (Fabregat et al., 2018; Jassal et al., 2020), and *KEGG* (Kanehisa et al., 2014, 2017). *STRING* contains all functional associations between molecules, including physical, or indirect but functional relations. Importantly, STRING associations are not assigned to any biological function in particular. *REACTOME* and *KEGG* are databases of pathways. However, interconversion and integration of both molecule IDs or biological functions is not easily done between the different databases. On the other hand, *Recon3D*, the most complete reconstruction of the human metabolic network, contains metabolic and transport reactions along with 3D structure of metabolites and proteins. Recon3D goes actually much further than the traditional definition of a *pathway* (as presented, for instance, in biochemistry textbooks and annotated in databases such as KEGG or Reactome) by introducing the so-called ReconMaps. Such ReconMaps are precisely-characterized depictions of human metabolic processes, including detailed information on the structural and functional, as well as spatial context (even at the organelle level), in which these processes (in the form of metabolic reactions and molecular interactions) occur. In this regard, Recon3D is presented as an unprecedented resource for future research to characterize biological functionality in humans.

In this work we built an integrated human network, including all metabolic reactions, protein-protein interactions, transcriptional regulation, transport and signaling processes based on KEGG database. We analyzed its topological properties and associated them to function when possible. We translated this molecular interaction network into a pathway network and studied how molecular perturbations translated into the disaggregation of the system. We studied different types of perturbations and applied several statistical and topological tests based on randomized null models. We found that the system is very resilient to specific types of molecular perturbations. This resilience could be caused by the presence of a complex, highly interdependent network connectivity structure based on the phenomenon of pathway crosstalk. We advance that further studies, along these lines, making use of extensive and well-annotated biological databases will provide a powerful tool to gain insights on the biomolecular origins of human health and disease.

## 2. Results

### 2.1. A Catalog of Molecular Interactions

**Figure 1:**
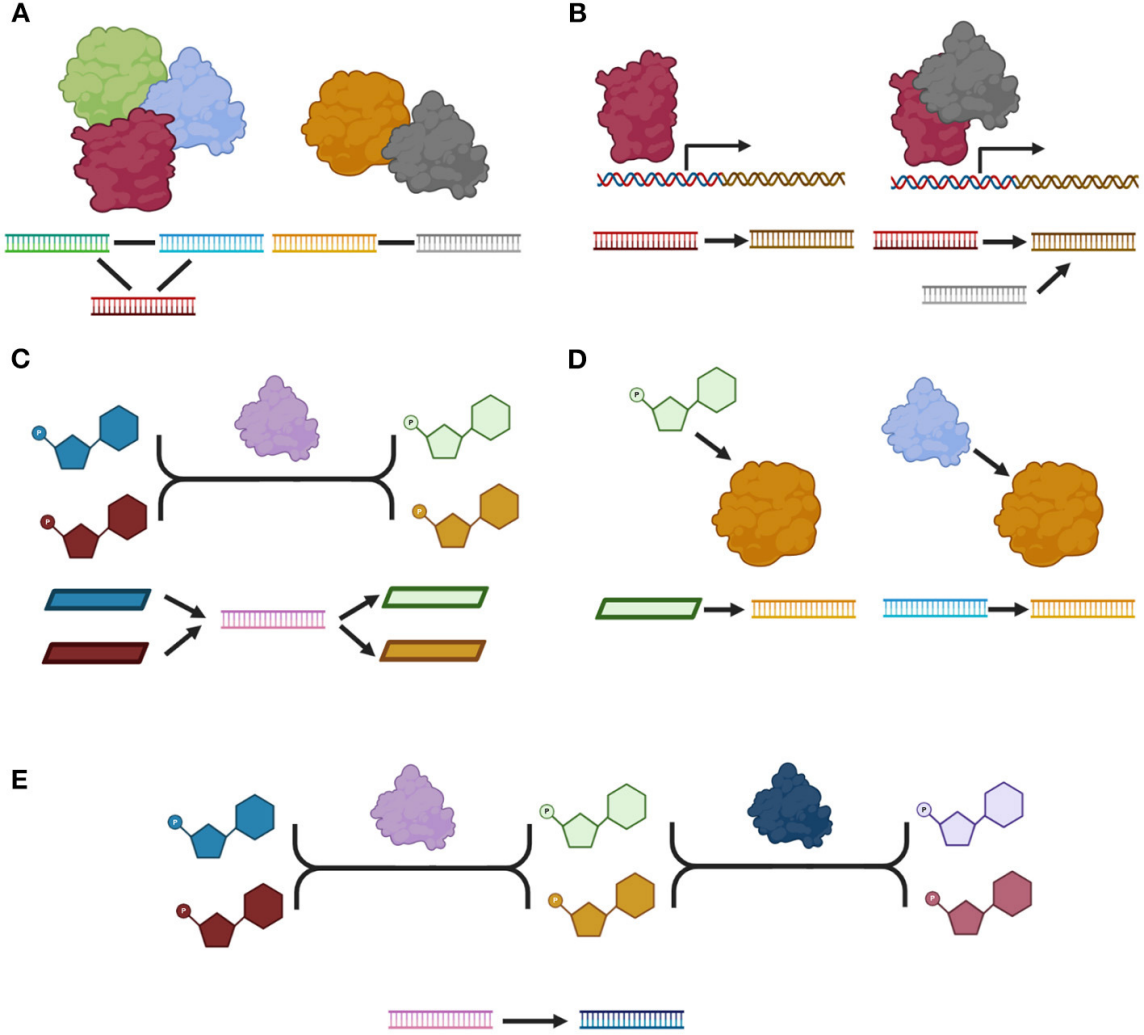
A catalog of molecular interactions. **(A)** Protein-protein interactions: right side, a protein-complex, left side, a transient physical interaction. **(B)** Regulatory interactions. **(C)** An enzymatic reaction. **(D)** Metabolic interactions. **(E)** Two consecutive reactions. We show a molecular model for each type and subtype of interaction as well as its representation in our network. All proteins are represented by their corresponding gene in our network. Proteins are depicted as by globular forms, genes by straight ladders and metabolites by polygons. A gene that codifies for a specific protein, is represented as a straight ladder of the same color as the corresponding globular form. Transcriptional regulation is represented by a square arrow. Arrows represent directed interactions and plain lines represent undirected interactions. Only the most common subtype of interactions are shown. Figure made using BioRender (Biorender.com).

Molecules can interact in different ways, resulting in a repertoire of biological responses. Protein-protein interactions (Figure 1A) occur when two proteins interact by establishing physical contacts between them; such contacts can be highly stable, giving rise to a protein complex or they could be transient to produce a specific short-term response. In a protein-protein interaction network (PPN), these interactions are represented by undirected connections between the genes that codify for such proteins. Another type of interactions, regulatory interactions, take place when a protein or a protein-complex (namely a Transcription Factor) regulates the expression of one or more target genes resulting in an increase or decrease in the activity of its target genes. In a regulatory network (RN) these interactions are represented by directed connections between the genes that codify the TF and the target genes (Figure 1B). At the metabolic level, metabolites are transformed by enzymes via metabolic reactions. The metabolic reactions are represented in a metabolic network (MN). Representations of such interactions include: bipartite graphs, in which directed connections are drawn from the substrates to the enzymes and from the enzymes to the products; and substrate graphs in which directed connections exist from any substrate to any product. In this work we will focus on the bipartite graph representation since it explicitly accounts for all molecules and we will represent each enzyme as the gene that codify for such enzyme (Figure 1C). In this work, we retrieved all these interactions from KEGG database. The most common subtypes of interactions are shown in Figure 1. Other not so common subtypes as well as the number of interactions per subtype are shown in Supplementary Figures 1–5.

Interestingly, there are two other types of interactions. Metabolic interactions between metabolites and proteins when a metabolite is known to affect the activity of a protein; or between two proteins if one of them has a post-translational effect over the activity of the other. The effect associated with these interactions could be any of the following: activation, inhibition, binding/association, missing interaction when the interaction is known to disappear due to mutation, dissociation, state change when the interaction represents a state transition, indirect effect and unknown. In this work, we did not include interactions cataloged either as indirect or unknown. Finally, KEGG contains a last type of interaction: two enzymes involved in consecutive reactions. Most of these interactions are redundant with the annotated enzymatic reactions. In this work, we only included the interactions for which there was no direct path between the participating enzymes via one metabolite in the metabolic network.

In this work we will start by analyzing three different types of very well-defined biological networks: *protein-protein interaction network* (PPN), *metabolic network* (MN), and *transcriptional regulatory network* (TRN). Later, we will construct an integrated network of metabolism by merging all isolated networks and including the additional interactions previously described. This is a dimensionality reduction as several layers of biological processes will be compacted. So, the processes behind a TF protein, A, that is transcriptionally regulating a target gene, B, and that forms a protein complex, AB, will be represented by only one interaction A interacts with B. In a similar way, if protein A is annotated to interact with protein B, and protein A is also annotated to post-translationally alter the activity of protein B, both relationships will be merged into the connection A interacts with B.

### 2.2. Analysis of Isolated Networks

We analyzed three different types of very well-defined biological networks: *protein-protein interaction network* (PPN), *metabolic network* (MN), and *transcriptional regulatory network* (TRN). Each molecular network was built based on the explanation provided in the last section. Each network consisted of 3,918, 2,963, and 916 nodes, respectively; and 34,927, 10,427, and 3,652 edges, respectively. All networks presented a Giant Connected Component (GCC) composed of more than 90% of nodes and edges (Supplementary Table 1). A giant connected component is defined as a connected component of a given network that contains a significant fraction (more than 50 %) of the nodes of the network. With regard to the degree distribution of each network, we found that all of them followed a power law distribution as well as their respective GCCs. (Jeong et al., 2000; Yu et al., 2004; Ouma et al., 2018). The goodness of fit statistic is shown for each network in Supplementary Table 2. The power law best fit parameters for each network were obtained and are shown in Supplementary Table 3.

**Figure 2:**
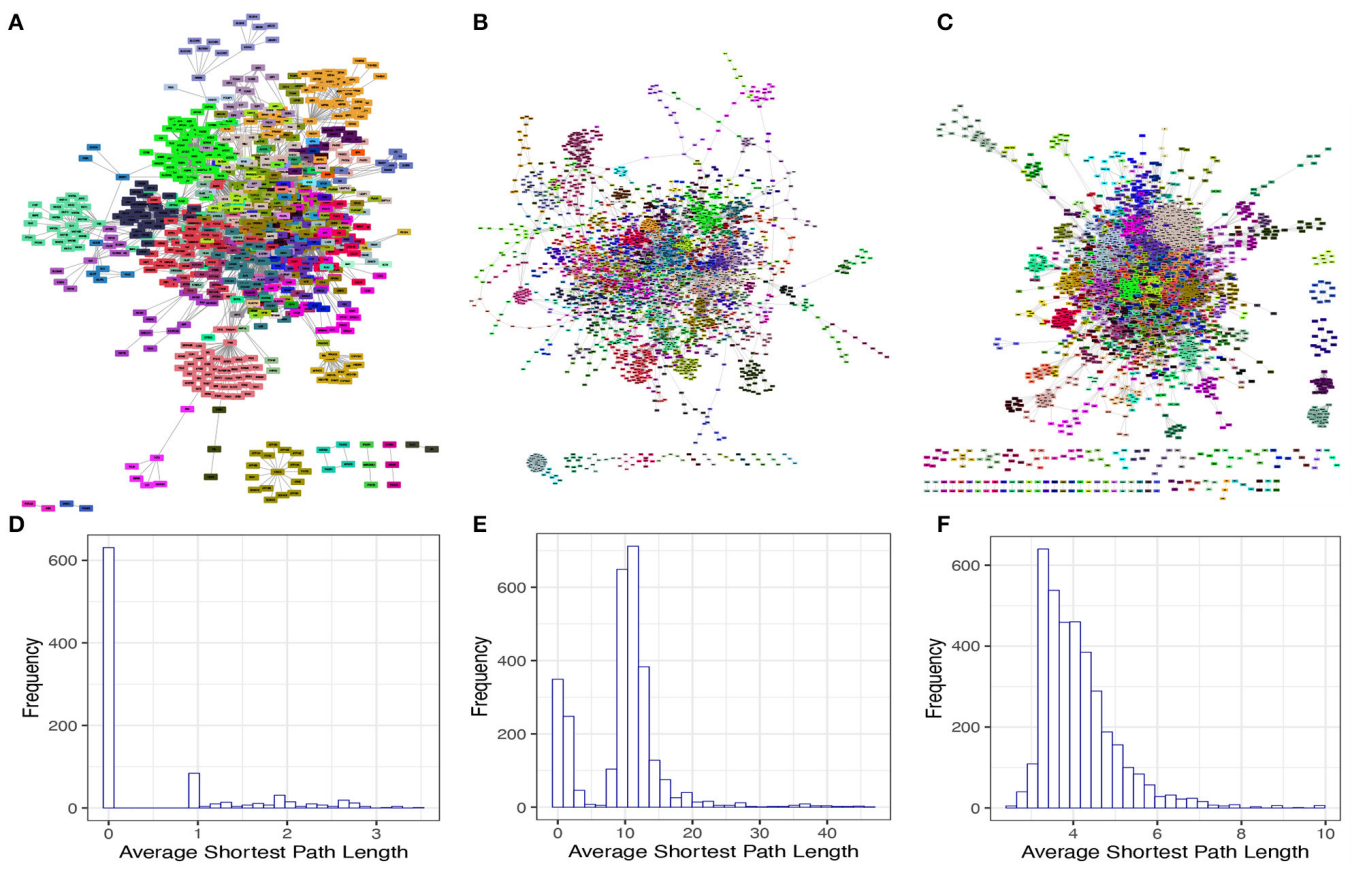
Modular structure and average shortest path length distribution of the isolated networks. **(A–C)** shows the modular structure for TRN, MN, and PPN, respectively. The color identifies to which module each node belongs to. **(D–F)** shows the average shortest path length distribution for the GCC of the TRN, MN, and PPN, respectively.

We next investigated some interesting topological and structural features in each of these isolated networks. We found that each of these networks has a statistically significant modular structure (Q=0.48 and *p* − *value* < 1*E*(−300) for the TRN; Q=0.79 and *p* − *value* < 1*E*(−300) for the MN; Q=0.68 and *p* − *value* < 1*E*(−300) for the PPN) (Figures 2A–C). We also found that the average shortest path length distribution behaves as This phenomenon can be explained by the directed nature of these networks as a considerable number of nodes present an in degree higher than zero but out degree equal to zero (Figures 2D–F).

### 2.3. The Estrogen Signaling Pathway as a Case Study

The aggregation of interactions from different biological layers in an integrated network offers the capability of looking at whole processes from a global perspective. As a proof of concept we show the estrogen signaling pathway (KEGG pathway: hsa04915).

Estrogens are a group of steroid hormones that have long been known as important regulators of the female reproductive functions, but also participate in the regulation of skeletal homeostasis, lipid and carbohydrate metabolism, electrolyte balance, skin physiology, the cardiovascular system, and the central nervous system (Vrtačnik et al., 2014). Estrogen mediates its cellular actions through multiple mechanisms of estrogen signaling, namely as *nuclear-initiated steroid signaling* and *membrane-initiated steroid signaling*. In the *nuclear* pathway, the estrogen binds to nuclear receptors, which in turn translocate to the nucleus, and interact directly with chromatin at specific DNA sequences known as estrogen response elements (EREs), acting as a transcription factor. On the other hand, in the *membrane* pathway, estrogen receptors, or G-protein coupled E2 receptors (GPER) found in the membrane can exert their action through the activation of second messenger proteins to relay the estrogen signal and exert physiological changes (Fuentes and Silveyra, 2019). Furthermore, estrogen signaling is also tightly connected with other important regulatory entities, i.e., epigenetic mechanisms, histone modifications, microRNAs, and DNA methylation (Vrtačnik et al., 2014).

**Figure 3:**
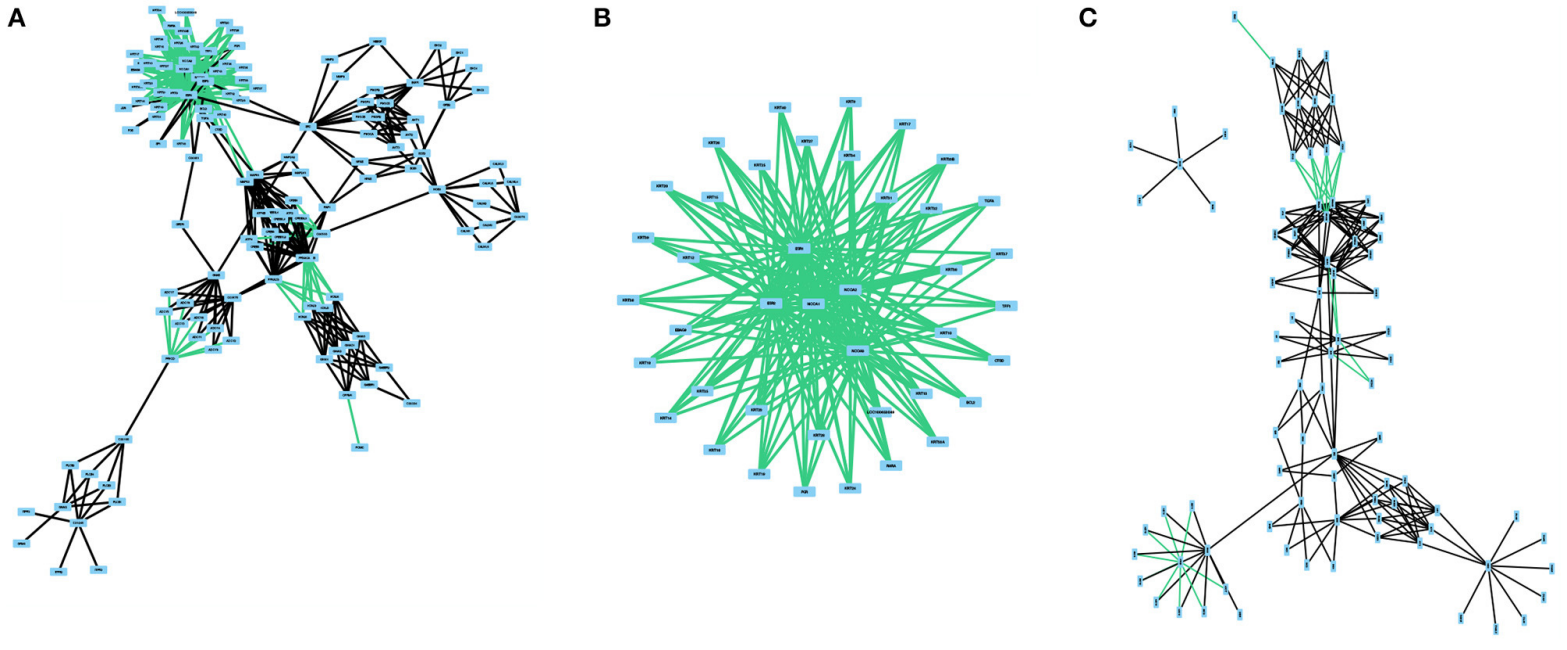
The different subnetworks related to estrogen signaling, regulation and protein interactions. All networks were obtained from KEGG. **(A)** presents the integrated network with all three network classes. **(B)** represents the regulatory network. **(C)** depicts the protein-protein interaction network. No catalytic reactions are present in the KEGG pathway.

The regulatory network and the protein-protein interaction network from this pathway are composed of 41 nodes and 180 edges and 87 nodes and 206 edges, respectively (Figure 3). Interestingly, no catalytic reactions are annotated as part of this pathway. And so, this pathway has no metabolic network. The integrated network for the estrogen signaling pathway is composed of 133 nodes and 433 edges. We investigated the crosstalk between the integrated network for the estrogen signaling pathway and any other human pathway. Crosstalk between two pathways exist if they share at least one molecule. We found crosstalk with the steroid hormone biosynthesis pathway, inositol phosphate metabolism pathway, butanoate metabolism pathway, purine metabolism pathway, alanine, aspartate, and glutamate metabolism pathway, arginine and proline metabolism pathway, and phosphatidylinositol signaling system. The complete set of these connections is not represented in any of the isolated networks and could only be studied in an integrated network such as the one built on this study.

### 2.4. A Comprehensive Network of Human Metabolism

**Figure 4:**
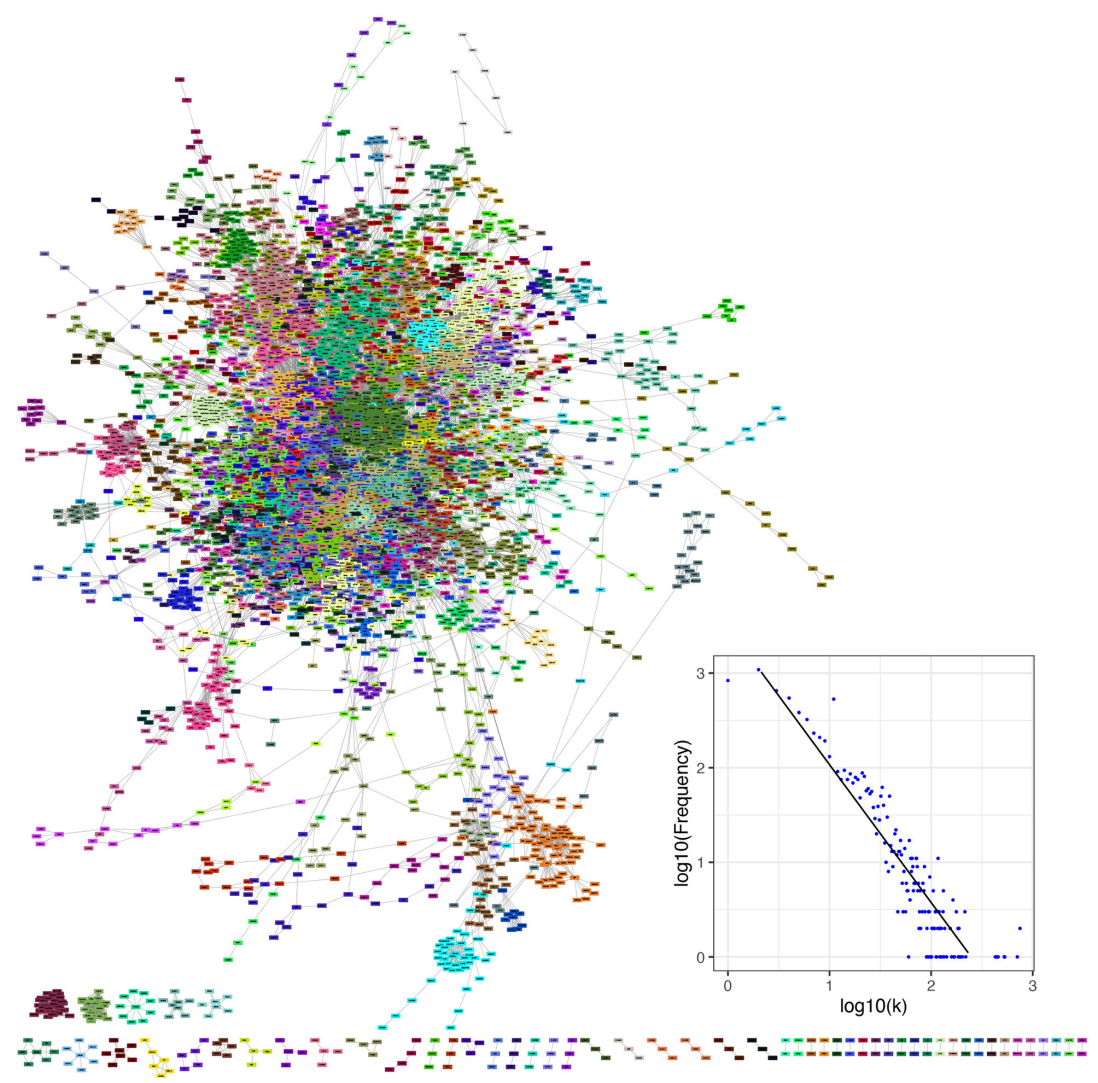
The human metabolism molecular interaction network the whole set of molecular interactions reported in the KEGG database for humans. It consists of 7,123 non-isolated biomolecules and 49,378 interactions (of diverse types) among them. Network communities are depicted by nodes of the same color. The inset shows the degree distribution in log-log scale for KEGG metabolism network.

In this study, we integrated all types of molecular interactions in a comprehensive network of human metabolism. We built an undirected network of human metabolism, including all molecular interactions reported in the KEGG database (Kanehisa and Goto, 2000; Kanehisa et al., 2014). We included all interactions contained in any isolated network. Additionally, there are some molecular interactions that are not incorporated in any formalism and that were also included in our integrated network, e.g., the relation between two enzymes catalyzing consecutive reactions and the metabolic interaction between a compound and a protein when the interaction is not part of an enzymatic reaction (Supplementary Figures 4, 5). Our network is composed of 10,676 nodes (biomolecules) and 49,378 edges (interactions). Non-interacting nodes were not taken into account for further calculations because they are usually not included in topological measures and they do not participate in pathway crosstalk (3,553 nodes) (Figure 4). The degree distribution of our integrated network (Figure 4) follows a power-law distribution with α = 3.17 and σ = 0.13 (Supplementary Table 4).

### 2.5. Modularity and Community Structure

Modular structure implies that a network can be divided into modules (also called communities). Network modules are loosely defined as subnetworks formed by sets of nodes (or vertices) that are more densely connected among themselves than with the rest of the network. It is generally believed that such semi-autonomous (but not independent) components of a network are responsible for *functionality* in real-life networks (Girvan and Newman, 2002; Newman and Girvan, 2004; Riolo and Newman, 2020). Often this functionality may be traced back to semi-mechanistic and/or statistical explanations. Such is the case of the statistical enrichment analysis performed in this work (Methods).

**Figure 5:**
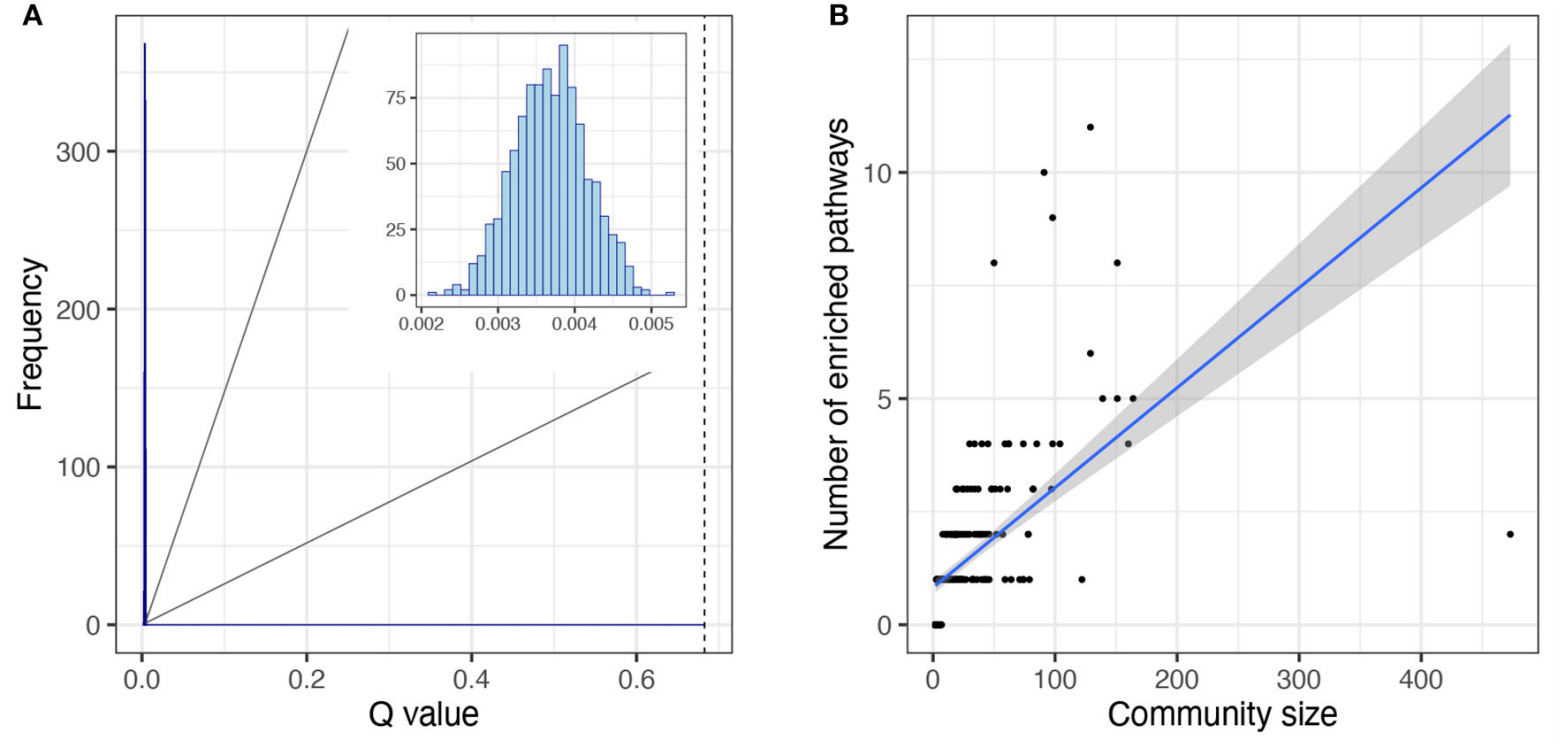
Topological features of modular structure of the human metabolism network. **(A)** Newman's Q distribution for the null models is shown in the histogram inset. The null models were obtained from community label reshuffling, 1,000 realizations. The dashed line represents the Newman's Q value obtained from the network of the human metabolism. **(B)** Correlation between module size and number of enriched, non-redundant pathways. The linear regression is shown as well as the confidence intervals [0.95].

We determined the community or modular structure of the human metabolism network using the Infomap algorithm (Rosvall and Bergstrom, 2007, 2008; Rosvall et al., 2009) and we calculated Newman's modularity coefficient, Q (see Methods). The significance of the modular structure was measured by random reshuffling of the module labels (1,000 realizations). We found that the network of human metabolism has a highly modular structure (Figure 5A), *Newman*′*sQ* = 0.68 [*p* − *value* < 1*E*(−300)].

We investigated whether the modular structure was related to function, in which case we could expect each module to be enriched in one or several related pathways. To investigate this phenomenon we obtained the non-redundant enriched pathways per module (see Methods). Notably, most modules showed enrichment for one or more non-redundant pathways (247 out of 321 modules) and the ones that did not present any enrichment had very few elements (Figure 5B). Pathways are defined by the annotations in biological databases such as KEGG. Annotations are based on empirical evidence (sometimes extremely detailed and refined and sometimes not) of biomolecular interactions between molecules associated with biological functions or phenotypes. A pathway is defined as the database annotation of a set of nodes. Some elements may or may not belong to the same module. A module is said to be *enriched* for a given pathway if it includes more nodes of the pathway than may be expected by chance alone (Rivals et al., 2007; Huang et al., 2009).

**Figure 6:**
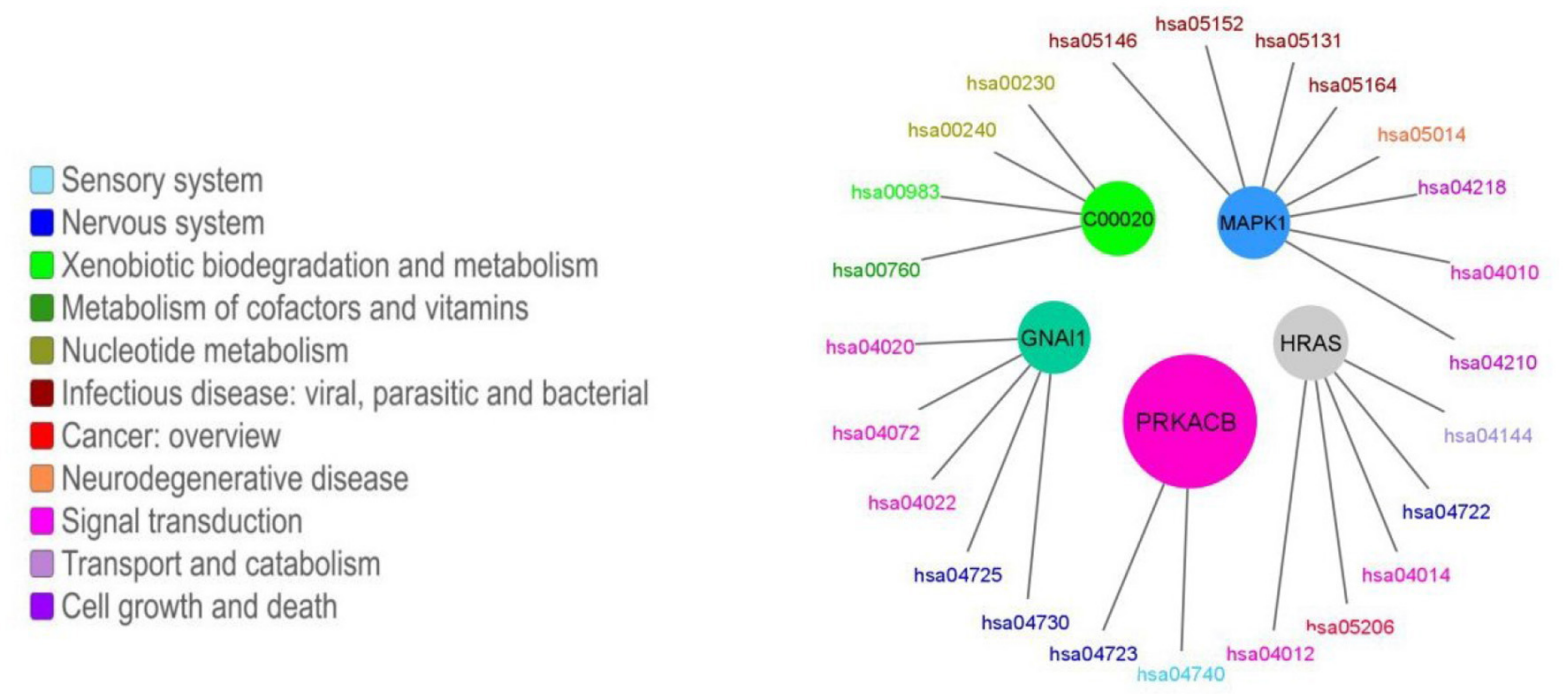
Enriched pathways for the five biggest modules. The colored circles represent each of the five biggest modules. The size of the circle is proportional to the number of nodes in each module. Each module is connected to its enriched pathways. The color of the pathways is related with their function, which was taken from KEGG's functional classification. Different tones of blue are associated with organismal systems, different tones of green are associated with metabolic pathways, different tones of red and orange are associated with human diseases, different tones of pink and purple represents the broad functional category of environmental information processing. Each module is named as one of the molecules it contains.

More than half of the modules presented enrichment for only one pathway (163 out of 247, 66%). Moreover, in the cases in which there is an enrichment for more than one pathway, there tends to be a functional relationship between the enriched pathways. The functional enrichment for the five biggest modules is shown in Figure 6. We can observe how the module that contains the GNAI1 gene is enriched in several nervous system pathways and signal transductions. By other hand, the module that contains the metabolite C00020 presents statistical significant enrichment in almost exclusively pathways labeled as metabolic. The information for all enrichments can be found in Supplementary File 1. Nevertheless, we found a correlation between the size of the community and the number of enriched pathways [ρ = 0.83, *p* − *value* < 2.2*E*(−16)] (Figure 5B). We observed the same tendency after repeating the analysis with the 95% trimmed distribution of the data [ρ = 0.35, *p* − *value* < 2.2*E*(−16)].

As a clear exception, we found that the biggest module (476 nodes) is enriched in only two pathways, this community is mostly devoted to the olfactory transduction pathway (418 nodes). This is unsurprising, since it is widely known that there is a plethora of olfactory receptors whose functions and structure are quite similar (Zozulya et al., 2001).

### 2.6. Topological and Structural Features of the Giant Connected Component (GCC)

**Figure 7:**
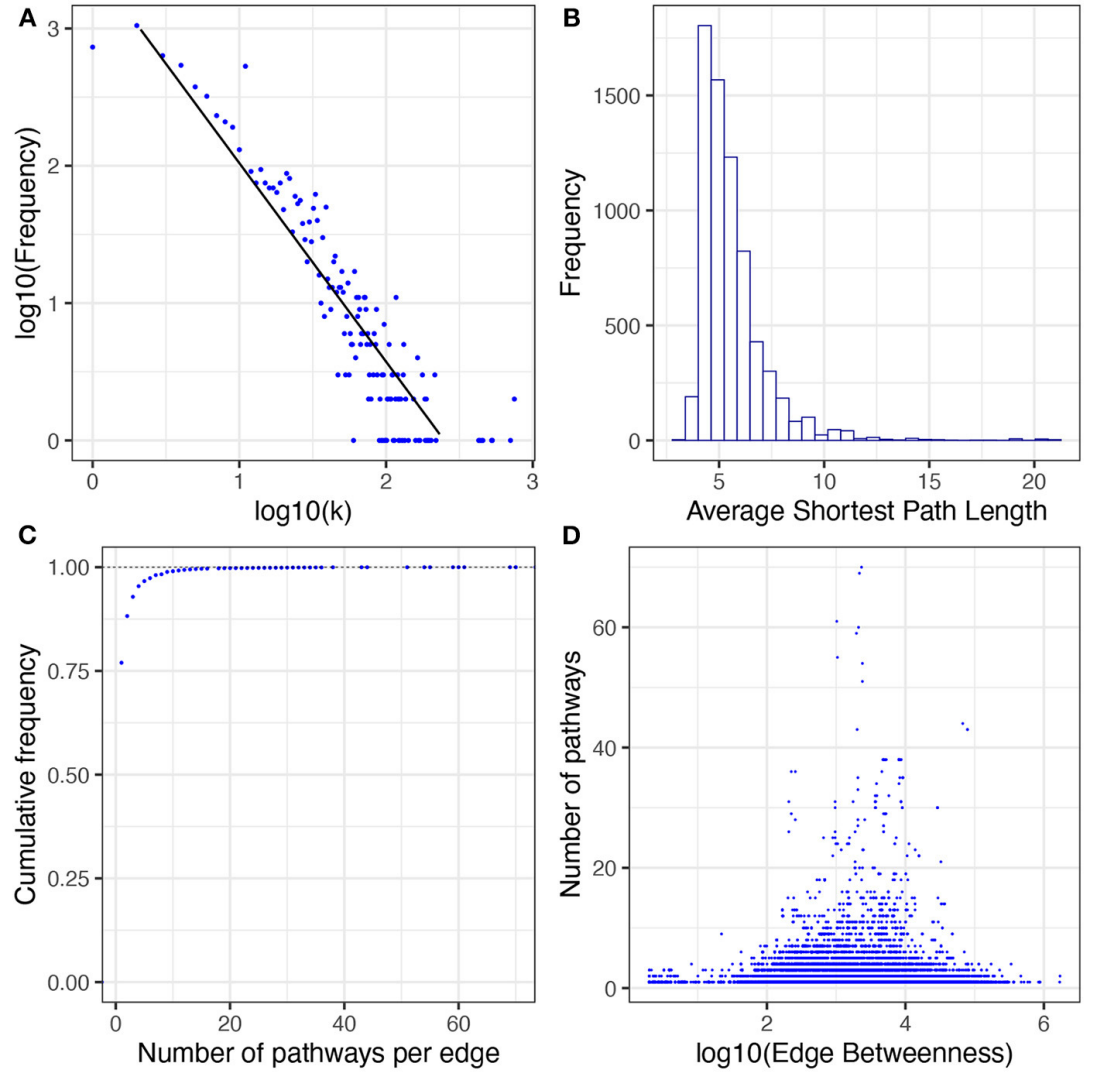
Topological and structural features of the GCC. **(A)** presents the degree distribution. **(B)** presents the average shortest path length distribution. **(C)** presents the cumulative distribution of pathways per edge. **(D)** presents the correlation between number of pathways and edge betweenness; a dot for each edge of the GCC is plotted. The x-axis corresponds to its value of edge betweenness and the y-axis corresponds to the number of pathways in which that interaction exists. No correlation patterns are apparent [ρ = −0.09, *p* − *value* < 2.2*E*(−16)].

In the network of human metabolism 6,894 nodes (96.8% of the total connected nodes, 64.6% of all the annotated biomolecules) and 48,663 edges (98.6% of the total interactions) form a GCC. This means that the vast majority of interacting molecules (96.8%) and their interactions (98.6%) in the annotated human metabolism belong to a single interconnected (interdependent) component. As with the whole interconnected network, we investigated whether the degree distribution of the GCC follows a power law (Figure 7A). We found that a power law distribution with α = 3.17 and σ = 0.13 is the best fit for our data (Supplementary Table 4).

The shortest path length is defined as the shortest distance between any two given nodes in a network. This measure usually correlates with the speed of the information flow throughout any network. The distribution of the average shortest path length for the GCC is highly skewed to the left, almost all nodes can reach any other node in less than 8 steps (Figure 7B). This result indicates that the network of human metabolism is highly compact which could be indicative of rapid information processing throughout the whole GCC.

The edge betweenness statistic describes how relevant is an edge for communication in the whole network. An edge could be relevant (i) if it is shared between multiple pathways, or (ii) if it connects two or more central pathways in the network. In the GCC we found that 76.98 % of the edges belong to only one pathway and only 0.96% of the edges belong to more than 10 pathways (Figure 7C). However, we found that there is no linear correlation between edge betweenness and number of pathways per edge [Spearman correlation test ρ = −0.09, *p* − *value* < 2.2*E*(−16), see Figure 7D]. This could suggest that the edges more relevant to transfer information through the network are the ones that connect central pathways.

Finally, we investigated if the GCC has a core-periphery structure (Csermely et al., 2013). Core-periphery structure refers to a certain type of modularization of the network, in which we can distinguish *core nodes* which are densely interconnected within a single or at most a few *cores*, whereas the so-called *periphery nodes* are sparsely interconnected among themselves and with the main core or cores (Borgatti and Everett, 2000; Kojaku and Masuda, 2018; Tang et al., 2019).

The closeness centrality statistic measures how central is a node in a network. So, central and peripheral nodes should have distinctive closeness centrality values. We calculated the closeness centrality statistic for every node in the GCC. The closeness centrality approximately follows a normal distribution; the peak found at 0.27 corresponds to the nodes annotated as olfactory receptors (> 400 nodes). We conclude that there is no evident core-periphery structure as the distribution is fundamentally unimodal. The core-periphery structure would be evidenced by the presence of two well-differentiated modes in the closeness centrality distribution (Supplementary Figure 6).

### 2.7. Redundancy and Resilience Defined From a Pathway Perspective

Genetic redundancy exists when there are two or more genes that perform the same molecular function, and so the inactivation of one of these genes has little or no effect on the phenotype (Nowak et al., 1997). Genetic redundancy produces biological resilience. From a system perspective, a system is resilient if it continues to function even in the face of external perturbations (Ungar, 2018). In this work, we investigated functional redundancy and metabolic resilience from a pathway perspective. We defined functional redundancy as the existence of more than one crosstalk between two pathways and metabolic resilience as the capacity of the system to continue to communicate between pathways even in the presence of perturbations.

Biological pathways communicate to each other via pathway crosstalk (Vert and Chory, 2011; de Anda-Jáuregui et al., 2019). This communication can happen when two pathways share molecules such as genes or metabolites. In this work, we built a pathway network (Supplementary Figure 7). We connected two pathways if they share at least one molecule. The resulting *pathway network* is formed by 293 pathways and 13,654 pathway crosstalk interactions. This network is extremely dense with an average degree of 93 and a network diameter of 6.

We will investigate if human metabolism presents functional redundancy, which could be translated into metabolic resilience to perturbations. From the molecular point of view, this resilience could be derived from node redundancy, i.e., two molecules that exist in any two pathways, or from edge redundancy, i.e., the existence of multiple interactions present in any two pathways which could bridge over any loss of function. From the global perspective, a metabolic process may exhibit resilience if it can recover from perturbations at the pathway level.

### 2.8. Analysis of Pathway Network

In order to analyze the extent of *resilience* of the human pathway network to perturbations in the metabolism molecular interaction network, we performed a percolation analysis (see Methods). The molecular network was perturbed by removing either (i) edges ordered by descending values of edge betweenness, (ii) nodes ordered by descending values of node degree, and (iii) nodes chosen at random. For each iteration, we created the corresponding pathway network and calculated the number of components, the number of edges, the mean degree, the number of nodes (pathways), and the average shortest path length. All these statistics were calculated taking into account only non isolated pathways.

**Figure 8:**
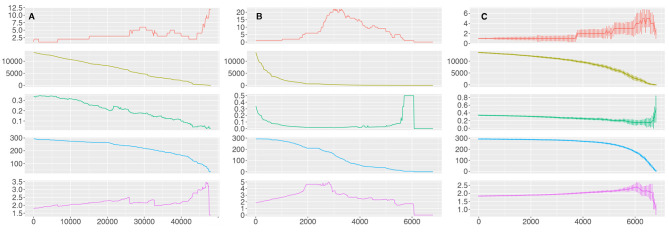
Percolation of the GCC. The results for the percolation analysis removing edges ordered by descending values of edge betweenness **(A)**, nodes ordered by descending values of node degree **(B)** and nodes chosen at random **(C)**. Each plot shows either the number of edges removed **(A)** or nodes removed **(B,C)** from the molecular interaction network in the X axis and the number of components, the number of edges, the mean degree, the number of nodes (pathways) and the average shortest path length of the pathway network in the Y axis for each iteration (top to bottom).

We can observe (Figure 8) that the number of edges in the pathway network (pathway crosstalk) mirrors the number of edges in the molecular interaction network; it shows either a linear decrease or an exponential decrease when 100 edges sorted by edge betweenness are removed or when 20 nodes sorted by degree are removed, respectively. However, we can observe that the network is quite stable trough the removal of edges sorted by relevance. Removal of the first 20,000 and first 40,000 top edge-betweenness molecular links (õne third and $\tilde{\text{t}}$wo thirds of the molecular links) results in the isolation of only 29 and 129 pathways, from the pathway network, respectively (9.9 and 44% of the pathways). There is a sharp structural breakdown, reminiscent of a phase transition, at around 45,000 removed edges (i.e., when $\tilde{9}$0% of the edges have been removed) as indicated by the drastic increase in the number of components and the severe increase in the average shortest path length.

This behavior is also seen when we remove nodes at random; the pathway network is broken into more than two components until around 5,000 nodes (out of 7,123, 70%) have been removed, implying that the pathway network is very resilient to the removal of random nodes at the molecular level. However, this behavior is dramatically switched when we remove nodes ordered by degree. In this case the number of pathways included in the pathway network decreases linearly and the network collapses when 2,000 nodes (28%) have been eliminated, as shown by the increase in the number of components and the increase in the shortest path length. At the point when 3,000 nodes have been removed the pathway network has been broken into more than 20 components and when 6,080 nodes have been removed there is no interacting pathway.

This result indicates that the pathway network is highly redundant and resilient to random failures at the node level and even to *targeted* perturbations at the edge level; however it is vulnerable to *targeted* perturbations at the node level.

To test for sensitivity of our results a null model was built by edge swapping keeping the connectivity distribution fixed. From these analyses we can see that the general trends, reported on the resilient behavior of the KEGG-based network, are still present (Supplementary Figure 14). To account for the effects of different connectivity distribution and different size we built three additional null models. For each null model we constructed the pathway network and performed a percolation analysis. We tested a random Erdös-Renyí network the same size as our KEGG network, a scale-free Barabási-Albert network 1.5 times as big as the KEGG network and a scale-free Barabási-Albert network 2 times as big as the KEGG network. We can observe that, in general, our results are robust and not-present in the null models although a size-effect can be noticed (Supplementary Figures 15–17).

## 3. Discussion

Metabolism is composed of a series of highly intertwined processes in which different types of biomolecules interact and react with each other. The study of the integrated network of the metabolism is important to shed light into relevant aspects about the structural organization and information transfer across this network.

Our study integrates for the first time the three types of biological networks in human. This integration allows the study of a more complete network of human metabolism, as it is evidenced by the estrogen signaling pathway example. In this example only a fraction of the processes of the pathway is represented in each isolated network and at least eight crosstalk between the estrogen signaling pathway and the human metabolism could not be observed if only isolated networks were studied.

In a network, modules (also known as communities) are groups of nodes characterized by a higher number of interactions between them than with any other group of nodes. In biological networks, modules have been associated with functional units, in particular with protein-complexes or dynamic functional units such as signaling cascades or cell-cycle regulation loops (Ravasz et al., 2002; Spirin and Mirny, 2003). We found that, in the case of the integrated network of human metabolism, modules are also related to function. More than half of the modules show functional enrichment for only one pathway. Moreover, if a module presents a significant enrichment for several pathways, they tend to be functionally related.

The shortest path length is commonly used as a measure of information flow efficiency across a network (Ye et al., 2010). Biological networks have been previously described as small-world networks for its connectivity distribution approximately follows a power law (Jeong et al., 2000; Yu et al., 2004; Ouma et al., 2018). Due to its small-world nature, the average shortest path length is expected to be quite small compared to the number of nodes in the whole network and it is expected to slowly increase as a function of the number of nodes in the network. In the case of a comprehensive, experimentally-validated, human protein-protein interaction network composed of 1,613 nodes, the mean shortest path length was 4.85 (Stelzl et al., 2005). On the other hand, genomic data was used to reconstruct the metabolic networks for 80 different organisms; the size of these networks ranged from 200 to 1,000 nodes. The average shortest path length for each domain of life was 9.57, 8.50, and 7.73 for eukaryota, archea, and bacteria, respectively (Ma and Zeng, 2003). In our integrated network composed of 7,123 nodes we found a mean shortest path length of 5.6. This number is similar to previous reports in protein-protein interaction networks and is lower than the one reported for metabolic networks. However, this is a low estimate when the number of nodes of the network is accounted for. The average distance between genes has been proposed as a proxy for functional relatedness. However, some studies have shown that the average shortest path length between genes associated with a specific disease is imposed by the degree of the chosen genes and it is independent of their function (Embar et al., 2016).

Edge betweenness is defined as the number of shortest paths between nodes that run along each edge (Girvan and Newman, 2002). If a high number of shortest paths go trough an edge that edge will have high edge betweenness (central edges). In a network, these central edges usually represent bridge-like connectors between different parts of a network, and they are the most efficient way to transfer information between different regions of the network. The removal of the most central edges has been applied to identify the modular structure of biological networks (Dunn et al., 2005; Yoon et al., 2006). In metabolism, a reaction or interaction between two molecules could be central because that interaction is present in a high number of pathways bridging all molecules between such pathways or because that central edge connects pathways that are central in the metabolism. We found that edge betweenness is not related to the number of pathways per edge what suggests that the edges more relevant to transfer the information through the network are the ones that connect central pathways.

Resilience is an intrinsic feature of biological systems as living organisms must respond to perturbations efficiently. Functional redundancy is often used to reach such stability. This phenomenon can be achieved by different genes performing the same function or by extensive crosstalk between pathways which bridges the lack of any specific molecule. The proportion of genes that can be deleted from an organism without producing a growth-rate defect has been found to vary considerably depending on the gene's pathway. In a massive study looking for growth defects produced by GAL1 promoter-driven over-expression only 15% of targets conferred a detectable growth defect (Sopko et al., 2006), while 25–30 and 76% of target genes in the case of the cell-cycle pathway and the HOG-pathway produced a growth retardation phenotype (Moriya et al., 2006; Krantz et al., 2009). On the other hand, some studies have found extensive crosstalk between pathways. In a study involving all the pathways that crosstalk with the estrogen signaling pathway, 1,400 out of 3,217 molecules involved in crosstalk events were shared by more than two pathways (de Anda-Jáuregui et al., 2015). However, how perturbations at the molecular level are translated into the pathway network has not been previously studied.

The integration of all network types sums up additional information in the construction of the pathway network as we observed in the estrogen signaling pathway. In this example, the number of pathways found in the pathway network ranges from 71, when only the metabolic network is considered, to 196 when only the protein-protein interaction network is considered. This implies that, in the best case, the crosstalk between only 67% (196 out of 293) of the total pathways could be studied if only one isolated network is analyzed.

Finally, our results indicate that the pathway network is highly redundant and resistant to random perturbations at the node level in the molecular network and even to *targeted* perturbations at the edge level in the molecular network. However, the pathway network is quite vulnerable to *targeted* perturbations at the node level in the molecular network. These results suggest that the best target candidate to break down the pathway network, disconnecting specific pathways, would be the most connected molecules. The minimum number of nodes that should be removed to isolate a specific pathway can be obtained from the pathway network built on this study. We firmly believe that detailed knowledge of the full topological and functional structure of the human metabolism network will improve our understanding of the basis of resilience and short-term adaptive processes to unprecedented levels, and will also open new, systemic approaches to molecular therapeutics.

## 4. Materials and Methods

### 4.1. Data Acquisition and Curation

Interaction and functional equivalence data was downloaded from the KEGG database via the KEGG API. Only human pathways were included in the analysis (*N* = 317, excluding the pathway *metabolic pathways*). Molecules and interactions were retrieved from the *kgml* files, no orthologs were included. Molecules with functional redundancy were retrieved from the *conf* files. All molecules were translated to NCBI Symbol and ENSEMBL ID using the NCBI Gene database and ENSEMBL Biomart for GRCh38.p12.

### 4.2. Reconstructing KEGG's Human Metabolism Interaction Network and the Biological Relevant Networks

A *kgml* file describes all the interactions and reactions form a particular pathway. It contains all enzymatic reactions indicating the substrate, the product and the enzyme involved in each reaction, as well as several types of relations between biomolecules: (1) relations between enzymes that catalyze consecutive reactions, (2) protein-protein interactions, (3) relations between a transcription factor and its target genes; (4) protein-compound interactions and (5) protein complexes. All these interactions were retrieved from each *kgml* file using a *custom-made* Python script. Types and subtypes of interactions were defined based on KEGG relation and object classification. If two molecules were described to be functionally redundant by the corresponding *conf* file, all the interactions associated with the first molecule were duplicated and assigned to the second molecule. In this way, an undirected network was created with either metabolites or genes as nodes and protein-protein, regulatory and metabolic interactions as edges. Each interaction was labeled with the list of pathways in which it appears.

To create the biological relevant networks only specific types of interactions were kept. TRN: only relations between a transcription factor and its target genes; MN: only enzymatic reactions and PPN: protein-protein interaction and protein-complexes.

### 4.3. Topological and Statistical Analyses

Community analysis was performed using the map equation algorithm optimizing a two-level partition of the network and including self links. The modularity coefficient was calculated using Newman's Q defined by:

$$Q=\frac{1}{2m}\underset{vw}{\sum }\left[A_{vw}-\frac{k_{v}k_{w}}{2m}\right]\delta \left(c_{v},c_{w}\right)$$

where *m* is the number of edges, *A*_*vw*_ is the element of the *A* adjacency matrix in row *v* and column *w*, *k*_*v*_ is the degree of *v*, *k*_*w*_ is the degree of *w*, *c*_*v*_ is the type (or component) of *v*, *c*_*w*_ that of *w*, and δ(*c*_*v*_, *c*_*w*_) is 1 if *v* = *w* and 0 otherwise. The null model was created by permuting the community labels (*N* = 1,000). Significantly enriched pathways per community were obtained by using Fisher's exact test.

The Cytoscape Network Analyzer package was used to calculate the undirected degree and the closeness centrality per node, the shortest path length between every pair of nodes and the edge betweenness per edge from the metabolism network.

To assess if the degree distribution follows a power law. We obtained the goodness of fit of our data to power-law, exponential, lognormal and truncated power distributions (Clauset et al., 2007; Alstott and Bullmore, 2014). We evaluated the goodness of fit to the power law distribution by comparing it to the fit to any other contending distribution.

### 4.4. Definition of Redundant Pathways and Enrichment Test per Community

An enrichment test was performed to find the significantly enriched pathways per module. The total number of elements per module and per pathway was calculated. For each module, all the pathways represented by at least one node were obtained and a Fisher's exact test was performed to test the significance of the enrichment. Two pathways were labeled as redundant if more than 70% of the molecules of the smallest pathway were contained in the biggest one. This threshold was chosen since it represents a good compromise between diminishing pathway redundancy, but keeping biological insight (Supplementary Figures 8–13). In the case of the redundant pathways only the biggest one was kept for further analysis. A Bonferroni correction was used to correct by multiple testing.

### 4.5. Analysis of Pathway Network

We built a pathway network from the molecular network. The pathway network was built by connecting two pathways if any of the following two conditions are fulfilled: (i) the two pathways have one (or more) shared nodes (molecules), or (ii) the two pathways have one (or more) shared edges. In order to perform the percolation analysis, we considered the whole set of edges in the network ordered by descending values of edge betweenness. Percolation analysis is performed by:

1. Removing 100 top edge betweenness interactions from the molecular interaction network
2. Recomputing network parameters: number of connected pathways, number of interactions, number of network components, mean degree, average shortest path length
3. Repeating.

The molecular network was perturbed by removing either (i) 100 edges ordered by descending values of edge betweenness, (ii) 20 nodes ordered by descending values of node degree, and (iii) 50 nodes chosen at random (30 realizations).

The networkx package in Python was used to build null model networks (Hagberg et al., 2008). The double_edge_swap function was used for edge swapping; the number of edges in our KEGG network was taken as the number of swaps, and the number of tries was taken as ten times the number of edges; 20 random networks were created. The gnm_random_graph function was used to create a Erdös-Renyí network; the number of nodes and edges were set to equal the number of nodes and edges in our KEGG network; 10 random networks were created. The barabasi_albert_graph function was used to create a scale-free Barabási-Albert network. The number of nodes was set to equal either 1.5 or 2.0 times the number of nodes in our KEGG network. The number of edges to be created for each additional node was set such that the number of edges in the final network was either 1.5 times or 2 times the number of edges in our network. 3 random networks of each type were created. For each of these molecular networks, a pathway network was created and a percolation analysis was performed as described previously.

### 4.6. Data Availability

We have uploaded the resulting network to our Github repository https://github.com/CSB-IG/KEGG-IntegratedNetwork along with the corresponding source code.

### 4.7. Glossary

We have included a glossary in the Supplementary Information.

## 5. Scope and Limitations

### 5.1. Annotations and Data Completeness

The present study is based on an exhaustive analysis of the molecular interactions reported in the Kyoto Encyclopedia of Genes and Genomes (KEGG) database, a highly curated and well annotated data resource for pathway annotation. It is worth-mentioning, however, that it does not mean *complete*. And that all the results and conclusions derived from analyzing this (or any other currently available) database it is contingent on incomplete annotation. In other words, for many phenomena (for instance, the fact that there are some small components on our network of human metabolism that are not connected to the main giant connected component of the network), the absence of evidence does not imply necessarily evidence of absence.

As previously discussed, an additional limitation of this work is that KEGG is one of several database resources for the annotation of metabolic and biomolecular cellular processes. Other databases are broader (such as STRING), larger (such as REACTOME) or focused (Recon3D). However, the rationale for using KEGG is that its interactions are more strictly curated than STRING or REACTOME (in fact, STRING uses KEGG annotations as its *gold standard* for validated interactions; Szklarczyk et al., 2019), and contains interactions aside from the purely metabolic. In the case of Recon3D, as we have mentioned, it makes a much more detailed depiction of biological function than that of *pathways*, as usually understood. Indeed, the level of detail of the ReconMaps is beyond that of any pathway/bioprocess databases in humans. For instance, Recon3D presents spatial and contextual compartmentalization information that goes beyond the scope of our present work here. By construction, the KEGG database in which this work is founded, does not inherently divide biomolecules into cellular compartments or components.

This is, indeed, one of the biggest shortcomings of KEGG as compared with the detailed, context-specific depictions used by approaches such as Recon3D. In this sense, we can view it as if it was a single compartment. However, interactions are mapped according to the contexts, or instances, in which they happen. For instance, metabolic processes located in the cell membrane, as described in KEGG, map molecules such as ion channels, ligands, receptors, and their interactions together, even though no explicit mention of their actual location or structure is given, in contrast with the detailed ReconMaps of Recon3D. Hence, within the scope of this work, no explicit compartmentalization is considered. As these other databases keep on developing, it may become relevant to further include them in analysis such as the one presented here.

We can observe that in general our results are robust and not-present in the null models (see the *Analysis of pathway network* section), although a size-effect can be noticed that, while does not invalidate our results, highlight the point about how progressive annotation of the databases in the future may affect the conclusions of our study.

### 5.2. Integrating Different Network Types

Regarding our integration of different networks related to metabolism, we have termed our approach a *network of human metabolism* instead of a *human metabolic network* to disambiguate between these two types of networks. Our rationale is as follows: distinguishing between metabolic pathways and other signaling, transcription and in general molecular pathways is conceptually useful to study some properties of metabolism such as biochemical kinetics and metabolic fluxes. It results, however, a little bit misleading if one seeks to understand the whole set of bioprocesses within an organism. Metabolic pathways are often triggered by signaling, both exogenous and endogenous and may involve transcriptional processes either upstream, downstream or within their metabolic activity course. A number of interactions involving protein complexes and molecular machines are needed to carry out metabolic (and signaling and transcriptional) responses.

In view of these facts, separating the different molecular networks by their type of elements, or by the directionality (or its absence) of their interactions, offers a partial view. A view that has been extremely useful and has been explored in the past with great success, to be fair. We have decided to start exploring ways to integrate these disparate sources of knowledge. We are aware that this work still represents an initial exploration of the issue. Proper mathematical methods to integrate diverse network types are currently being developed as well as frameworks to make sense of the biology of these integrated models. Some of these have to do with the so-called multi-omic approaches.

## Data Availability Statement

Publicly available datasets were analyzed in this study. This data can be found at: https://www.genome.jp/kegg/pathway.html.

## Author Contributions

EH-L and LG-R conceived the project. EH-L directed and supervised the project. LG-R designed and develop the computational strategy. LG-R and KL-R implemented the code and database search procedures, and conducted the calculations and validation. KL-R, LG-R, and EH-L analyzed the results. All authors read and approved the final manuscript.

## Conflict of Interest

The authors declare that the research was conducted in the absence of any commercial or financial relationships that could be construed as a potential conflict of interest.
