# Supplementary material for: The Large Scale Structure of Human Metabolism Reveals Resilience via Extensive Signaling Crosstalk
Source: Front Physiol. 2020 Dec 16;11:588012. doi: 10.3389/fphys.2020.588012 (PMC7772240; doi:10.3389/fphys.2020.588012)
Supplement: Supplementary file 1 [file Data_Sheet_2.pdf]

The large scale structure of human metabolism reveals resilience via extensive signaling crosstalk

SUPPLEMENTARY INFORMATION

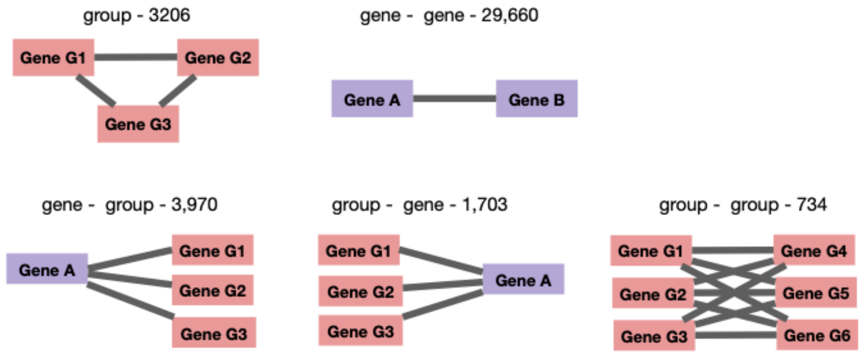

Figure S1. All different subtypes of protein-protein interactions included in KEGG database. The number of molecular interactions derived from each interaction subtype is shown. A group refers to a protein-complex (a stable interaction formed between two proteins). The interaction subtypes refer to the following: gene-gene: a transient physical contact between two proteins; gene-group: transient physical contact between a protein and a protein-complex; group-gene: transient physical contact between a protein-complex and a protein. In our undirected network gene-group and group-gene interactions are interchangeable. A group-group interaction refers to the transient physical contact between two protein-complexes.

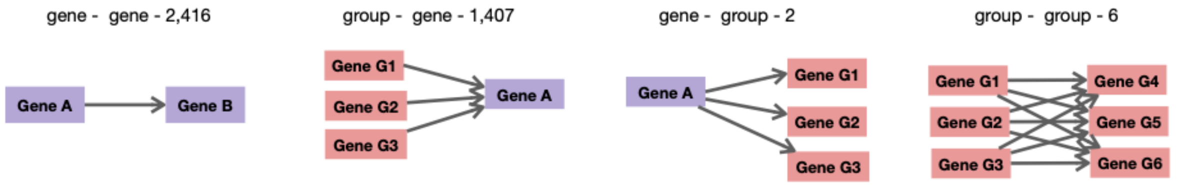

Figure S2. All different subtypes of regulatory interactions included in KEGG database. The number of molecular interactions derived from each interaction subtype is shown. A group refers to a protein-complex (a stable interaction formed between two proteins). The interaction subtypes refer to the following: gene-gene: a TF that regulates the expression of a target gene; group-gene: a protein-complex that regulates the expression of a target gene; gene-group: a TF that regulates the expression of the genes involved in a protein-complex; group-group: a protein-complex that regulates the expression of the genes involved in a protein-complex. The molecular direction of the interaction is indicated by the direction of the arrow.

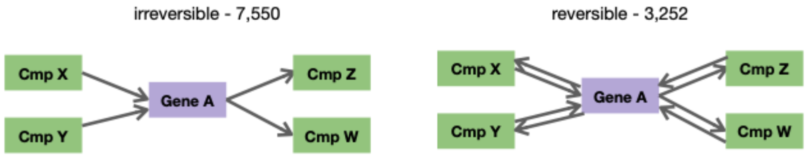

Figure S3. All different subtypes of interactions derived from enzymatic reactions included in KEGG database. In the left panel, the enzyme codified by geneA catabolized the enzymatic reaction in which metabolite X and Y are transformed into metabolite Z and W. In the right panel, the reverse reaction is catabolized by the same enzyme. The number of molecular interactions derived from each interaction subtype is shown. In total, there are 1416 genes and 1547 metabolites participating in enzymatic reactions. In the directed metabolic network, the interactions from a reversible reaction must account for both directions of the reaction

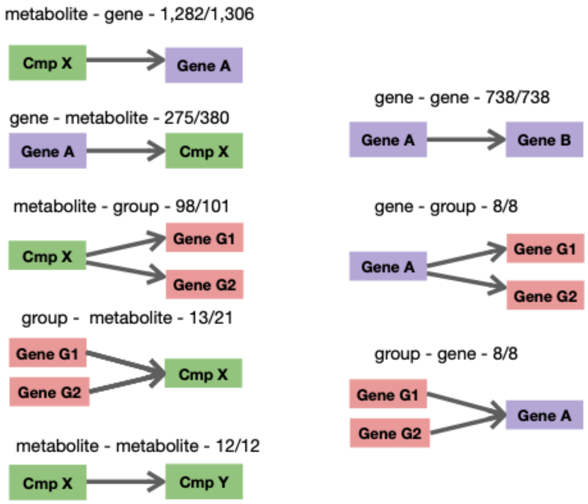

Figure S4. All different subtypes of interactions derived from metabolic interactions included in KEGG database. The number of molecular interactions derived from each interaction subtype is shown. A group refers to a protein-complex (a stable interaction formed between two proteins). The interaction subtypes refer to the following: metabolite-gene: a metabolite has an effect over the activity of a protein; gene-metabolite: the activity of a protein exerts an effect over the amount of a metabolite; metabolite-group: a metabolite has an effect over the activity of a protein-complex; group-metabolite: the activity of a protein-complex exerts an effect over the amount of a metabolite; gene-gene: a protein has a post-translational effect over the activity of another protein; gene-group: a protein has a post-translational effect over the activity of a protein-complex; group-gene: a protein-complex has a post-translational effect over the activity of a protein; group-group: a protein-complex has a post-translational effect over the activity of a protein-complex.The molecular direction of the interaction is indicated by the direction of the arrow. For each subtype of interaction the number of interactions included in our integrated network/the number of total interactions in KEGG database is shown.

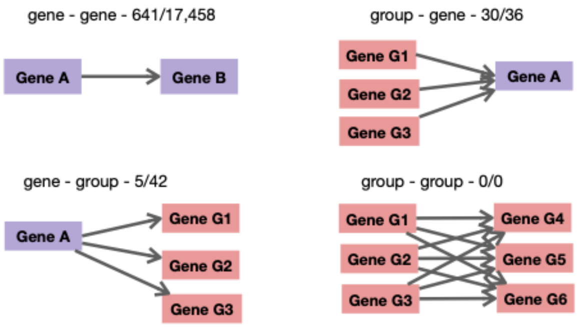

Figure S5. All different subtypes of interactions derived from the category of successive enzymatic reactions included in KEGG database. The number of molecular interactions derived from each interaction subtype is shown. A group refers to a protein-complex (a stable interaction formed between two proteins). The interaction subtypes refer to the following: gene-gene: two enzymes catalyze successive reactions; gene-group: an enzyme and a protein-complex catalyze successive reactions; group-gene: a protein-complex and an enzyme catalyze successive reactions. There is no annotation about two protein-complexes catalyzing successive reactions. The molecular direction of the interaction is indicated by the direction of the arrow. For each subtype of interaction the number of interactions included in our integrated network/the number of total interactions in KEGG database is shown.

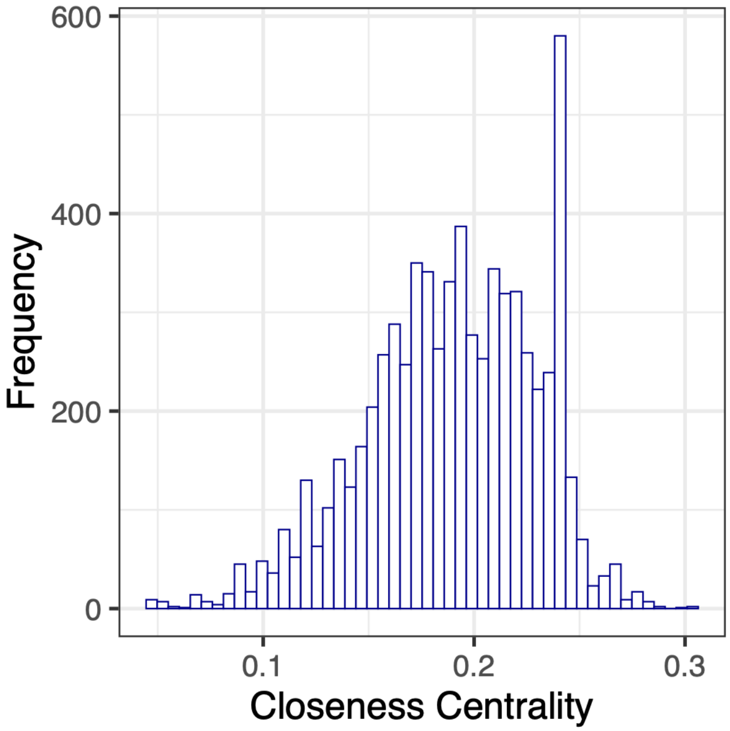

Figure S6. Closeness centrality of the GCC. No core-periphery structure is evident in the GCC network. Closeness centrality approximately follows a normal distribution, so there is no clear distinction between central and peripheral nodes.

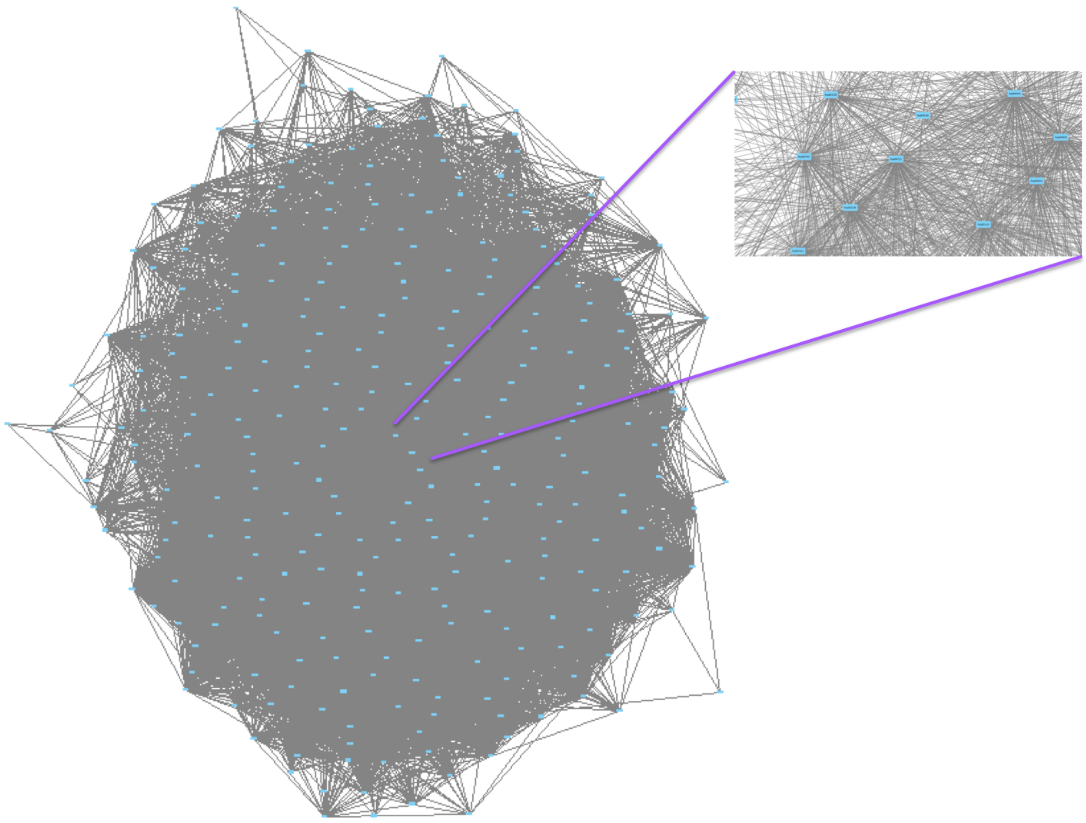

Figure S7. A network of human pathways: In this pathway crosstalk network, the nodes represent pathways and there exist an interaction between nodes when those pathways share at least one molecule. The inset shows how dense is this network (<k> = 103).

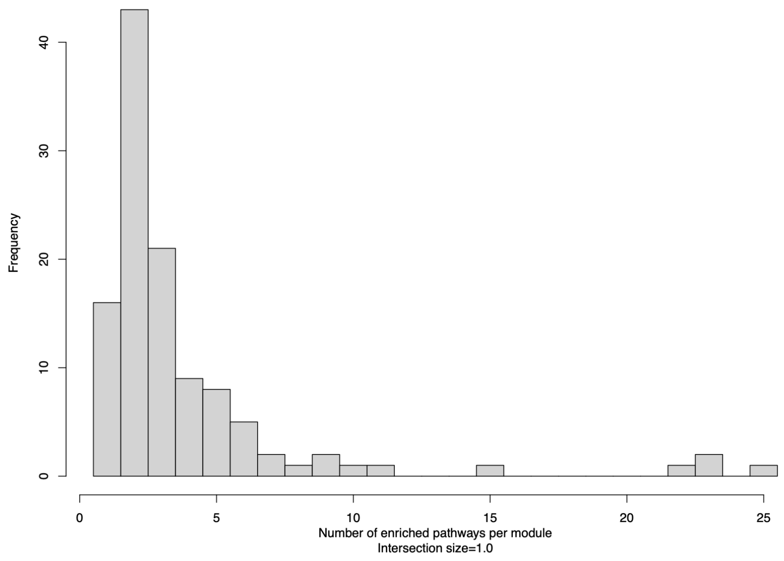

Figure S8. The distribution of number of non-redundant pathways enriched per module. The x- axis correspond to the number of non-redundant pathways enriched per module, the y-axis indicates the number of modules with that number of non-redundant enriched pathways. Two pathways are considered redundant if 100% of the elements of the smallest pathway are contained in the largest one

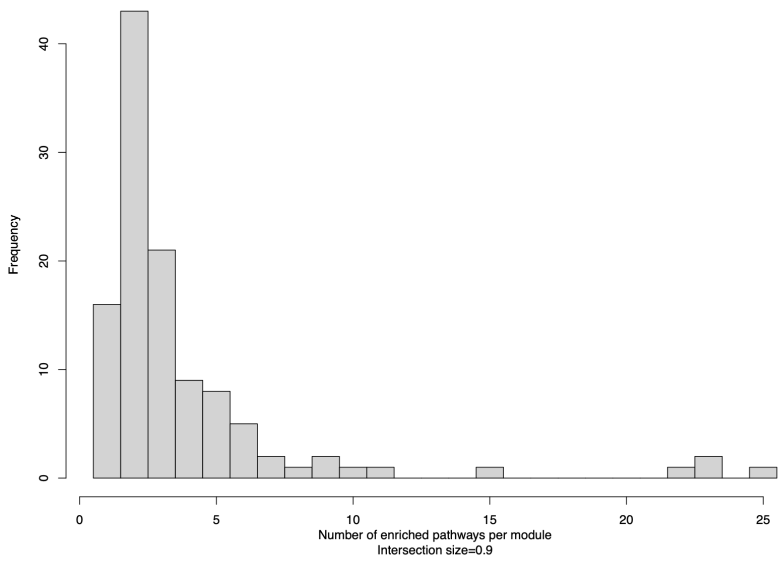

Figure S9. The distribution of number of non-redundant pathways enriched per module. The x- axis correspond to the number of non-redundant pathways enriched per module, the y-axis indicates the number of modules with that number of non-redundant enriched pathways. Two pathways are considered redundant if more than 90% of the elements of the smallest pathway are contained in the largest one.

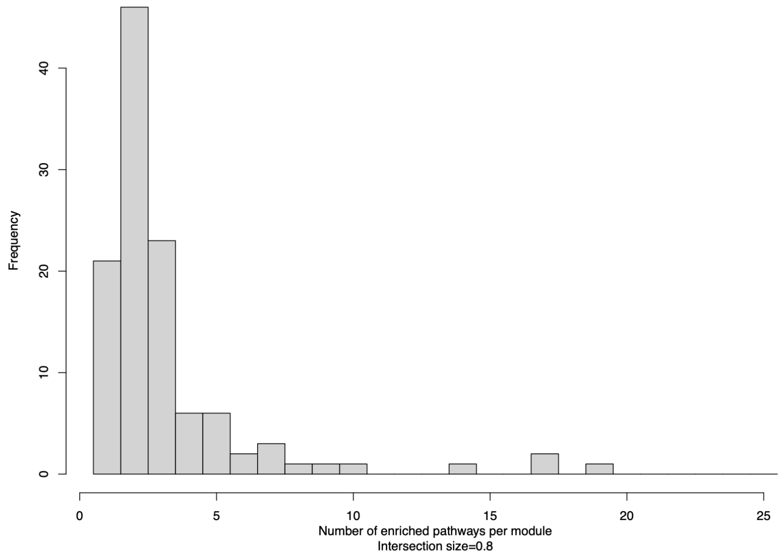

Figure S10. The distribution of number of non-redundant pathways enriched per module. The x- axis correspond to the number of non-redundant pathways enriched per module, the y-axis indicates the number of modules with that number of non-redundant enriched pathways. Two pathways are considered redundant if more than 80% of the elements of the smallest pathway are contained in the largest one

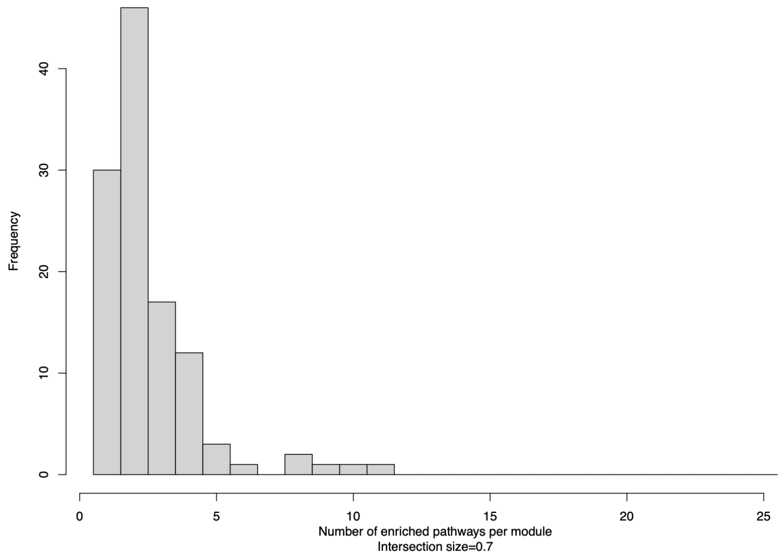

Figure S11. The distribution of number of non-redundant pathways enriched per module. The x- axis correspond to the number of non-redundant pathways enriched per module, the y-axis indicates the number of modules with that number of non-redundant enriched pathways. Two pathways are considered redundant if more than 70% of the elements of the smallest pathway are contained in the largest one.

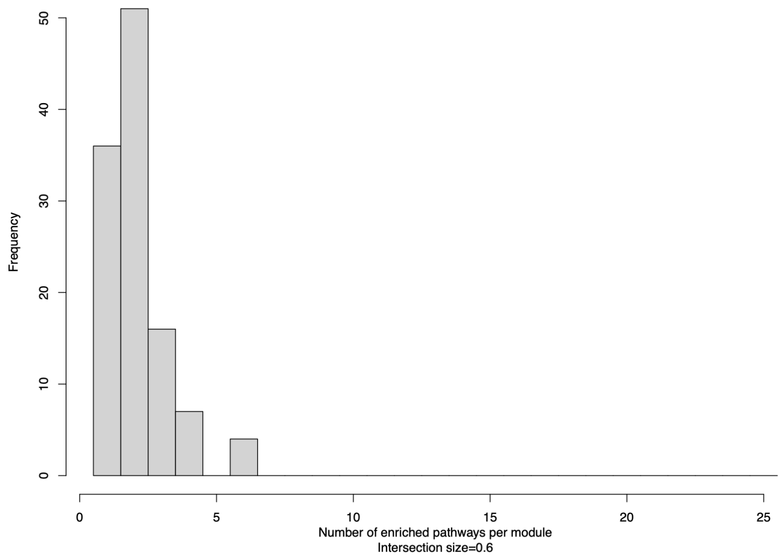

Figure S12. The distribution of number of non-redundant pathways enriched per module. The x- axis correspond to the number of non-redundant pathways enriched per module, the y-axis indicates the number of modules with that number of non-redundant enriched pathways. Two pathways are considered redundant if more than 60% of the elements of the smallest pathway are contained in the largest one

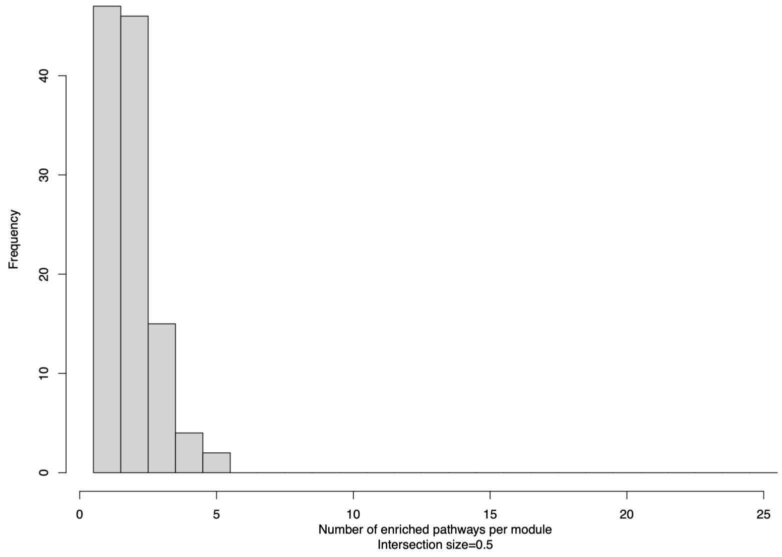

Figure S13. The distribution of number of non-redundant pathways enriched per module. The x- axis correspond to the number of non-redundant pathways enriched per module, the y-axis indicates the number of modules with that number of non-redundant enriched pathways. Two pathways are considered redundant if more than 50% of the elements of the smallest pathway are contained in the largest one.

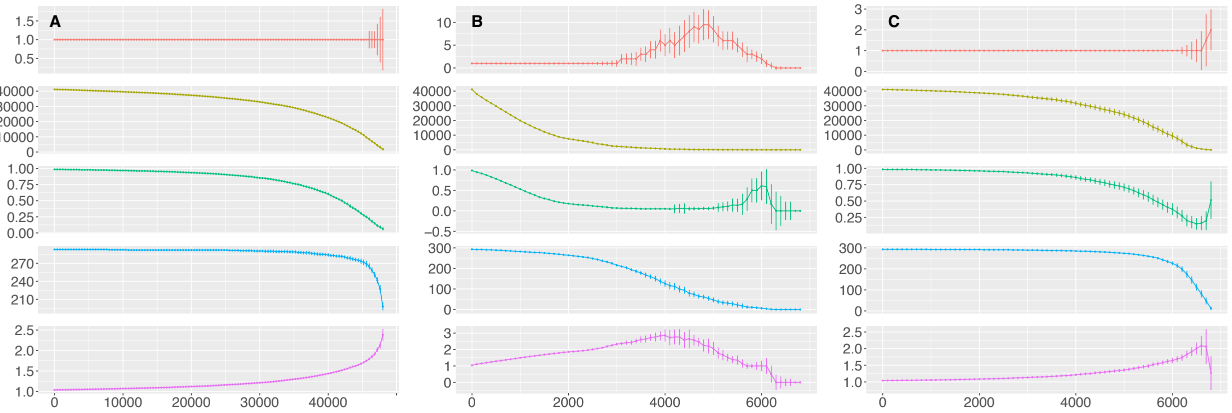

Figure S14. A null model was built by edge swapping from the original KEGG network keeping the connectivity distribution fixed. A pathway network and a percolation analysis were done from this null model. The results for the percolation analysis removing edges ordered by descending values of edge betweenness (Panel A), nodes ordered by descending values of node degree (Panel B) and nodes chosen at random (Panel C) are shown. Each plot shows either the number of edges removed (A) or nodes removed (B and C) from the molecular interaction network in the X axis and the number of components, the number of edges, the mean degree, the number of nodes (pathways) and the average shortest path length of the pathway network in the Y axis for each iteration (top to bottom)

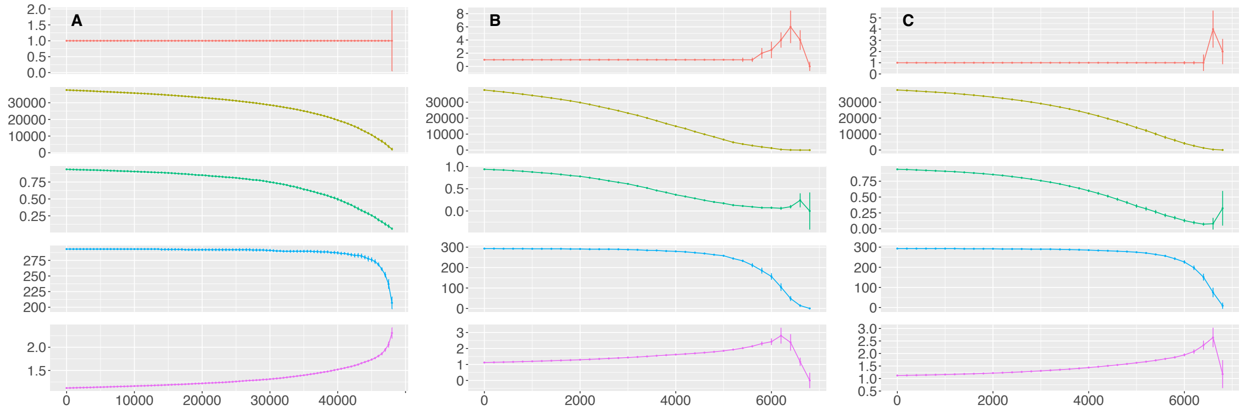

Figure S15. A random Erdös-Renyí network the same size as our KEGG network was built. A pathway network and a percolation analysis were done from this network. The results for the percolation analysis removing edges ordered by descending values of edge betweenness (Panel A), nodes ordered by descending values of node degree (Panel B) and nodes chosen at random (Panel C) are shown. Each plot shows either the number of edges removed (A) or nodes removed (B and C) from the molecular interaction network in the X axis and the number of components, the number of edges, the mean degree, the number of nodes (pathways) and the average shortest path length of the pathway network in the Y axis for each iteration (top to bottom).

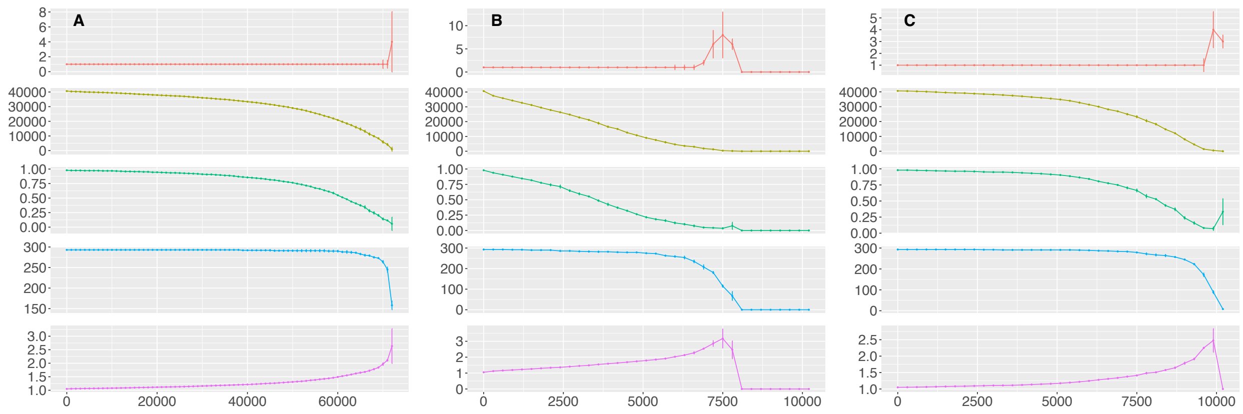

Figure S16. A scale-free Barabási-Albert network 1.5 times as big as the KEGG network was built. A pathway network and a percolation analysis were done from this network. The results for the percolation analysis removing edges ordered by descending values of edge betweenness (Panel A), nodes ordered by descending values of node degree (Panel B) and nodes chosen at random (Panel C) are shown. Each plot shows either the number of edges removed (A) or nodes removed (B and C) from the molecular interaction network in the X axis and the number of components, the number of edges, the mean degree, the number of nodes (pathways) and the average shortest path length of the pathway network in the Y axis for each iteration (top to bottom).

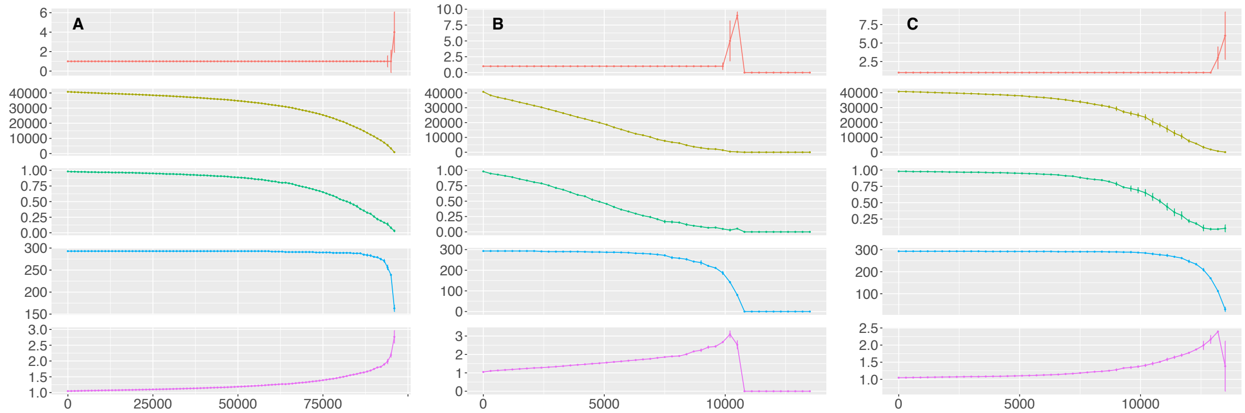

Figure S17. A scale-free Barabási-Albert network 2 times as big as the KEGG network was built. A pathway network and a percolation analysis were done from this network. The results for the percolation analysis removing edges ordered by descending values of edge betweenness (Panel A), nodes ordered by descending values of node degree (Panel B) and nodes chosen at random (Panel C) are shown. Each plot shows either the number of edges removed (A) or nodes removed (B and C) from the molecular interaction network in the X axis and the number of components, the number of edges, the mean degree, the number of nodes (pathways) and the average shortest path length of the pathway network in the Y axis for each iteration (top to bottom).

SUPPLEMENTARY TABLES

	TRN	MN	PPN
**Whole network**			
Number of nodes	916	2963	3918
Number of edges	3652	10427	34927
**GCC**			
Number of nodes	884	2862	3668
Number of edges	3626	10165	34191

Table S1. Number of nodes and edges in the whole network and in the GCC for the human TRN, MN and PPN.

	TRN		MN		PPN	
**DISTRIBU-TION**	**R**	**P**	**R**	**P**	**R**	**P**
Exponential	164.31	1.2E(-15)	248.86	9.0E(-23)	47.85	3.5E(-5)
Truncated power law	-0.71	0.235	-14.28	9.0E(-8)	1.2E(-8)	0.999
Lognormal	-2.49	0.042	-1.41	0.275	-2.49	0.016
Lognormal positive	18.69	2.9E(-5)	19.10	0.005	2.77	0.032

Table S2. The goodness of fit for the TRN, MN and its GCC and the PPN is shown. R is the log likelihood ratio between the two candidate distributions (power law distribution and the one in the corresponding row). This number will be positive if the data is more likely a power law distribution, and negative if the data is more likely to be attributed to the tested distribution. The significance value for that direction is p.

The statistics derived from the GCCs for the different networks are not shown since they behave practically identical to their complete network counterparts.

	α	σ
**TRN**	2.3	0.06
**MN**	2.35	0.03
**PPN**	3.18	0.14

Table S3. Power law best fit parameters for the human TRN, MN, MN GCC and PPN. The α and σ parameters for the GCCs are identical to the best fit parameters of the respective complete network.

**DISTRIBUTION**	**R**	**P**
Exponential	47.02	0.0003
Truncated power law	1.4E(-5)	0.99
Lognormal	-2.73	0.01
Lognormal positive	1.86	0.17

Table S4. R is the log likelihood ratio between the two candidate distributions. This number will be positive if the data is more likely a power law distribution, and negative if the data is more likely to be attributed to the tested distribution. The significance value for that direction is p. The goodness of fit for the whole network (removing isolated nodes) is shown.We the must notice that almost all of the network nodes and edges belong to Giant Connected Component (GCC) that actually shares the same statistics and will become a central study object in this work from now on.

SUPPLEMENTARY GLOSARY

- **Bipartite graph**: A network with two different types of nodes. These nodes form a disjoint set and every edge (or link) of the network connects nodes from different type. A bipartite graph does not contain odd-length cycles.
- **Community (see, Module)**
- **Component:** A network component, often called a connected component or island, in an undirected network is a subgraph (a part of the network) in which any two nodes are connected to each other by one or more paths, and which is connected to no additional nodes in the supergraph (the full network)
- **Degree centrality**: Degree centrality or simply degree is defined as the number of links incident on a node (i.e., the number of ties that a node has). The degree measures the flow of information through this node in the network. In the case of a directed network two separate degree centralities are defined, in-degree and out-degree. Accordingly, in-degree is a measure of the number of links directed to the node and outdegree is the number of links that such node directs to others
- **Edge Betweenness:** or Edge Betweenness Centrality is a centrality measure for the links, it is defined as the number of the shortest paths that go through a given link in a network
- **Enzyme graph:** In the context of the present paper an enzyme graph or enzyme network is an important biological network which relates enzyme proteins and chemical compounds. It is the graph theoretical depiction of a metabolic pathway or a series of metabolic pathways.
- **Giant Connected Component (GCC):** A giant connected component is a connected component of a given network that contains a significant fraction (more than 50 \%) of the nodes of the network
- **Hypergraph:** a hypergraph is a generalization of a network in which a link can connect to an arbitrary number of nodes. In contrast, in an ordinary network, a link connects exactly two nodes. In the context of this work KEGG hypergraphs have been “flattened” by multiplicating edges.
- **Metabolite interactions graph:** In the context of KEGG a metabolite interactions graph is different to an enzyme network in that it only contains metabolites as nodes
- **Module:** Network modules are understood as subnetworks formed by sets of nodes (or vertices) that are more densely connected among themselves than with the rest of the network. Modules are often viewed as semi-autonomous (but not independent) components of a network are responsible for functionality in real networks
- **Shortest path:** A shortest or geodesic path, between two nodes in a network is a trajectory with the minimum number of edges. If the network edges are weighted, it is a path with the minimum sum of edge weights. The length of a geodesic path is called geodesic distance or shortest distance. Geodesic paths are not necessarily unique, but the shortest path distance is well-defined since all shortest paths have the same length.
